# Supplementary material for: Clinical Potential of Kinase Inhibitors in Combination with Immune Checkpoint Inhibitors for the Treatment of Solid Tumors
Source: Int J Mol Sci. 2021 Mar 5;22(5):2608. doi: 10.3390/ijms22052608 (PMC7961781; doi:10.3390/ijms22052608)
Supplement: Supplementary file 1 [file ijms-22-02608-s001.pdf]

Table 1. Summary of kinase inhibitors approved by the FDA for treatment of solid tumors, their molecular targets and the studies demonstrating their regulatory roles on tumor immunogenicity or immune responses.

TKI	Target	Approved for	Immunomodulation
**CDK4/6 inhibitors**			
Abemaciclib	CDK4/6	BC	Increases antitumor immunity and synergizes with immunotherapy in CRC [1, 2], and in ES[3].
Ribociclib	CDK4/6	BC	Increases antitumor immunity [4]
Palbociclib	CDK4/6	BC	Increases immunogenicity in breast cancer [5] and synergizes with immunotherapy in bladder cancer [6]
**Alk inhibitors**			
Alectinib	Alk, fusion/L1196M	NSCLC	Increases PD-L1 expression in NSCLC [7], increases HLA [8]
Brigatinib	Alk, EGFR and their mutants	NSCLC	Unknown; was approved on May 22, 2020
Ceritinib	Alk, Alk fusion/point mutants	NSCLC	Altered immune responses, but did not alter immunogenicity to synergize with αPD-1 antibody in NSCLC mouse model [9]
Crizotinib	Alk, Alk fusion, c-Met	ALCL, NSCLC	Increases HLA, antigen presenting machinery [8] and increases T cell-APC interaction [10]. Synergizes with chemo and immunotherapy in mice model of NSCLS [11, 12]. Hepatoxicity observed when used with ICI [13]
Lorlatinib	Alk, Ros1	NSCLC	Unknown; was approved on November 2, 2018
Entrectinib	Trk, Ros1, Alk	NSCLC, ST	Unknown; was approved on August 15, 2019
**MEK inhibitors**			
Binimetinib	MEK1/2	MEL	Reviewed in [14]
Cobimetinib	MEK1	MEL	Reduces B regulatory cells in colorectal cancer [15]. Impedes apoptosis of chronically stimulated, antigen-experienced CD8+T cells in colon cancer model and synergizes with αPD-L1 antibody to have tumoricidal activity [16], but did not show increased survival benefit when combined with atezolizumab in patients [17]
Trametinib	MEK1/2	ATC, MEL, NSCLC	Reviewed in [14]. Partial inhibition of T cell function; increase CD8+ T cell infiltration with anti-PD-1 antibody in colon cancer line [18]. Increased HLA in melanoma [18]. Enhanced immune cell infiltration [19] and tumor control when combined with PD-1 blockade in melanoma mouse model [20, 21]
**RAF inhibitor**			
Dabrafenib	BRAF	ATC, MEL, NSCLC	Reviewed for melanoma [22]. Increases HLA in melanoma [18]. Doesn't impair healthy T cell function [23].
Encorafenib	RAF	CRC, MEL	Unknown; was approved on April 8, 2020.
Vemurafenib	BRAF(V600E)	ECD, MEL	Reviewed for melanoma [22]. Alters CD4+T cell function [24]. Suppresses MDSC [25]. Modulates both antitumor and protumor immunity in melanoma [19]. Enhances immunogenicity [26]
**EGFR family inhibitor**			
Afatinib	EGFR, HER2, HER4, EGFR L858R/T790M	NSCLC	Increases antitumor immunity in GC[27].
Dacomitinib	EGFR family	NSCLC	Unknown; was approved September 18, 2018
Erlotinib	EGFR	NSCLC, PC	Increases antitumor immunity [28], and synergizes with immunotherapy in colorectal cancer [29].

Gefitinib	EGFR	NSCLC	Alters immune responses in mouse lung cancer model [30]
Lapatinib	EGFR, ERBB2	BC	Increases antitumor immunity in gastric cancer [27] and IFNγ-driven antitumor adaptive immune response in breast cancer [31].
Neratinib	HER2, EGFR	BC	Increases MHC and decreases PD-L1, PD-L2 in vitro; with HDAC inhibitor, sensitizes to immunotherapy in mouse [32]
Osimertinib	T790M EGFR	NSCLC	Alters immune responses in lung cancer mouse model [30]
Tucatinib	HER2	BC	Unknown; was approved on April 17, 2020
Cetuximab	EGFR	CRC, SCCHN	Reviewed in [33]. Augments immune responses that are favorable for antitumor activity [34]
Margetuximab	HER2	BC	Elicits improved ADCC compared to trastuzumab [35] and high HER2-specific antitumor immune response [36]
Necitumumab	EGFR	SNSCLC	Induces ADCC; reviewed for NSCLC [37]
Panitumumab	EGFR	CRC	Elicits antitumor immune response, albeit at lower level than does cetuximab [38]
Pertuzumab	HER2	BC	Elicits antitumor immune responses, namely antibody-dependent cell-mediated cytotoxicity (ADCC) and phagocytosis (ADCP) by innate immune cells. Reviewed in [39]
trastuzumab*	HER2	BC	Elicits ADCC and ADCP; reviewed in [39].
**mTOR**			
Everolimus	mTOR	BC, PC, GIC, LC, RCC, SGCA.	Blocks T cell function and used as an immunosuppressant in organ transplantation [40, 41]. Associated with reduced CTL infiltration and increased T regulatory cells in prostate cancer [42]. Inhibits T cell and NK cell activity *in vitro* [43]. Using cyclophosphamide was shown to alleviate its immunosuppressive effects [44].
Temsirolimus	mTOR	RCC	Increases antitumor immunity and synergizes with anti-tumour vaccine in RCC and MEL [45]
**Others**			
Alpelisib	PI3Kα	BC	When combined with Ribociclib, enhances tumor immunogenicity *in vitro* and elicits T cell infiltration that's associated with reduced breast cancer growth in mouse [4]
Avapritinib	Kit mutant, PDGFRa D842V	GIST	Unknown; was approved on January 9, 2020
Axitinib	VEGFR1/2/3, Kit, PDGFR	RCC	Less adverse effects on T cell function compared to sorafenib *in vitro* [46]
Cabozantinib	c-Met, VEGFR1/2/3, FLT3, TIE2, TRKB, Axl, RET	HCC, MTC, RCC	Enhances antitumor immunity in colon cancer mouse model [47] and synergizes with tumor vaccine [48]
Capmatinib	MET	NSCLC	Suppresses T cells *in vitro*, upregulates PD-L1 in HCC cells; improves tumor killing when combined with αPD-1 antibody [49]
Erdafitinib	FGFR	UC	Synergizes with αPD-1 antibody to elicit antitumor immune response in lung cancer mouse model [50].
Imatinib	c-kit, PDGFR	GIST, MDS/MPN, SM	Antitumor activity depends on host immune response and synergizes with immunotherapy [51]; reviewed for GIST [52]. Modulates various immune cells in cancer (reviewed in [53]). Affects macrophage activity in vitro [54, 55]
Larotrectinib	Trk	ST	Unknown; was approved on November 26, 2018

Lenvatinib	VEGFR2	EMC, HCC, RCC, TC	Reviewed for HCC in [56]. Reduces tumor-associated macrophages, increases CTLs, and synergizes with αPD-1 antibody [57, 58]
Fedratinib	JAK2, FLT3	MF	Negatively regulates regulatory T cells and T helper cell cytokine secretion [59]. Downregulates PD-L1 expression in lung cancer [60] and lymphoma cell lines [61]
Pazopanib	PDGFR, VEGFR1/2/3, Kit	RCC, STS	Primes DC to confer antitumor immune response [62]
Pralsetinib	RET	TC, MTC, NSCLC	Unknown; was approved on December 1, 2020
Pemigatinib	FGFR1/2/3	CCC	Unknown; was approved on April 17, 2020
Pexidartinib	KIT, CSF1R, FLT3	TSGCT	Negatively affects tumor-associated macrophages, and synergizes with dendritic cell vaccination to eliminate mesothelioma in mouse model [63]. Reduces infiltration of immunosuppressive macrophages to improve immunotherapy [64]
Regorafenib	VEGFR2/3, Ret, Kit, PDGFR, Raf	CRC, GIST, HCC	Increases CTL driven antitumor immune response when used with αPD-1 antibody in HCC mouse model [65]. Reviewed in [56]. Modulates macrophage to enhance antitumor immunity; reviewed in [66]
Ripretinib	SCFR KIT, PDGFRa, VEGFR2, TIE2, CSF1R	GIST	Unknown; was approved on May 15, 2020
Ruxolitinib	JAK1/2	MF, PV	Is immunosuppressive and anti-inflammatory through affecting various immune cells (reviewed in [67]). Suppresses cytokine release syndrome while not impeding CART cell therapy in AML mouse model [68]. Enhances T cell dependent antitumor immune response and synergizes with αPD-1 antibody in pancreatic cancer mouse model [69].
Selpercatinib	RET, VEGFR1/3, FGFR1/2/3	MTC, NSCLC, TC	Unknown; was approved on May 8, 2020
Sorafenib	Raf, VEGFR2, PDGFRb	HCC, RCC, TC	Elicits antitumor immune response, regulates myeloid infiltrates and synergizes with cancer vaccine in colon cancer mouse model [70]. Adversely impacts T cell function *in vitro* [46]. Reduces regulatory T cell infiltration in RCC patients [71]
Sunitinib	PDGFR, FLT3 VEGFR1/2, Kit,	GIST, PC, RCC	Increases antitumor immunity [72-75] and reduces immunosuppression, synergizes with tumor vaccine in mouse model of colon cancer [70]
Vandetanib	VEGFR2, EGFR	MTC	Unknown; was approved on April 7, 2011
Ramucirumab	VEGFR2	CRC, HCC, NSCLC, SA, GEJA	Negatively regulates regulatory T cells, enhances CD8+ T cell infiltration and PD-L1 expression in gastric cancer patients [76]. Improves patient survival when used with pembrolizumab [77].

Source for drug, its target and designated cancer: National Cancer Institute. ↑ or ↓, increase or decrease in tumor intrinsic immunogenicity or association to increased antitumor immune responses. ▲or▼, increase or decrease in antitumor activity of immune cells directly impacted by the treatment. **ATC**, Anaplastic thyroid cancer. **ALL**, acute lymphoblastic leukemia. **ALCL**, anaplastic large cell lymphoma. **BC**, breast cancer. **CC**, cervical cancer. **CCC**, cholangiocarcinoma. **CRC**, colorectal cancer. **DFSP**, dermatofibrosarcoma protuberans. **ECD**, Erdheim-Chester disease. **EMC**, Endometrial cancer. **ES**, Ewing sarcoma. **GBM**, glioblastoma. **GC**, gastric cancer. **GIC**, gastrointestinal cancer. **GEJA**, gastroesophageal junction adenocarcinoma. **GIST**, Gastrointestinal Stromal Tumor. **HCC**, Hepatocellular carcinoma. **LC**, lung cancer. **LSCC**, lung squamous cell carcinoma. **MEL**, melanoma. **MF**, myelofibrosis. **MDS/MPN**, myelodysplastic/myeloproliferative neoplasms. **MTC**, Medullary thyroid cancer. **(S)NSCLC**, (Squamous) Non-small cell lung cancer. **OEC**, ovarian epithelial cancer. **FTC**,

fallopian tube cancer. **PPC**, primary peritoneal cancer. **PC**, pancreatic cancer. **PV**, Polycythemia vera **RCC**, renal cell carcinoma. **SA**, stomach adenocarcinoma. **STS**, soft tissue sarcoma. **SCCHN**, squamous cell carcinoma of the head and neck. **ST**, certain solid tumors. **SGCA**, subependymal giant cell astrocytoma. **SM**, systemic mastocytosis. **TC**, thyroid cancer. **UC**, Urothelial cancer. **TSGCT**, tenosynovial giant cell tumor. *with hyaluronidase-oysk, or as antibody drug conjugate Ado-Trastuzumab emtansine, or in combination with pertuzumab and hyaluronidase-zzxf. fam-trastuzumab deruxtecan-nxki is approved for BC, GC, GEJA

References

1. Schaer, D. A., R. P. Beckmann, J. A. Dempsey, L. Huber, A. Forest, N. Amaladas, Y. Li, Y. C. Wang, E. R. Rasmussen, D. Chin, A. Capen, C. Carpenito, K. A. Staschke, L. A. Chung, L. M. Litchfield, F. F. Merzoug, X. Gong, P. W. Iversen, S. Buchanan, A. de Dios, R. D. Novosiadly, and M. Kalos. "The Cdk4/6 Inhibitor Abemaciclib Induces a T Cell Inflamed Tumor Microenvironment and Enhances the Efficacy of Pd-L1 Checkpoint Blockade." *Cell reports* 22, no. 11 (2018): 2978-94.
2. Hurvitz, Sara A., Miguel Martin, Michael F. Press, David Chan, María Fernandez-Abad, Edgar Petru, Regan Rostorfer, Valentina Guarneri, Chiun-Sheng Huang, Susana Barriga, Sameera Wijayawardana, Manisha Brahmachary, Philip J. Ebert, Anwar Hossain, Jiangang Liu, Adam Abel, Amit Aggarwal, Valerie M. Jansen, and Dennis J. Slamon. "Potent Cell-Cycle Inhibition and Upregulation of Immune Response with Abemaciclib and Anastrozole in Neomonarch, Phase Ii Neoadjuvant Study in Hr⁺/Her2[−] Breast Cancer." *Clinical Cancer Research* 26, no. 3 (2020): 566-80.
3. Dowless, Michele, Caitlin D. Lowery, Terry Shackleford, Matthew Renschler, Jennifer Stephens, Robert Flack, Wayne Blosser, Simone Gupta, Julie Stewart, Yue Webster, Jack Dempsey, Alle B. VanWye, Philip Ebert, Philip Iversen, Jonathan B. Olsen, Xueqian Gong, Sean Buchanan, Peter Houghton, and Louis Stancato. "Abemaciclib Is Active in Preclinical Models of Ewing Sarcoma Via Multipronged Regulation of Cell Cycle, DNA Methylation, and Interferon Pathway Signaling." *Clinical Cancer Research* 24, no. 23 (2018): 6028-39.
4. Teo, Zhi Ling, Stephanie Versaci, Sathana Dushyanthen, Franco Caramia, Peter Savas, Chris P. Mintoff, Magnus Zethoven, Balaji Virassamy, Stephen J. Luen, Grant A. McArthur, Wayne A. Phillips, Phillip K. Darcy, and Sherene Loi. "Combined Cdk4/6 and Pi3kα Inhibition Is Synergistic and Immunogenic in Triple-Negative Breast Cancer." *Cancer Research* 77, no. 22 (2017): 6340-52.
5. Goel, S., M. J. DeCristo, A. C. Watt, H. BrinJones, J. Sceneay, B. B. Li, N. Khan, J. M. Ubellacker, S. Xie, O. Metzger-Filho, J. Hoog, M. J. Ellis, C. X. Ma, S. Ramm, I. E. Krop, E. P. Winer, T. M. Roberts, H. J. Kim, S. S. McAllister, and J. J. Zhao. "Cdk4/6 Inhibition Triggers Anti-Tumour Immunity." *Nature* 548, no. 7668 (2017): 471-75.
6. Long, Q., A. H. Ma, H. Zhang, Z. Cao, R. Xia, T. Y. Lin, G. P. Sonpavde, R. de Vere White, J. Guo, and C. X. Pan. "Combination of Cyclin-Dependent Kinase and Immune Checkpoint Inhibitors for the Treatment of Bladder Cancer." *Cancer immunology, immunotherapy : CII* 69, no. 11 (2020): 2305-17.
7. Shimada, Masahiro, Atsuhisa Tamura, Kyoko Yokosuka, Kei Kusaka, Hirotoshi Matsui, Hideaki Nagai, and Ken Ohta. "A Successful Pembrolizumab Treatment Case of Lung Adenocarcinoma after Becoming Resistant to Alk-Tki Treatment Due to G1202r Mutation." *Respiratory Investigation* 56, no. 4 (2018): 365-68.
8. Oh, Claire Y., Martin G. Klatt, Christopher Bourne, Tao Dao, Megan M. Dacek, Elliott J. Brea, Sung Soo Mun, Aaron Y. Chang, Tatyana Korontsvit, and David A. Scheinberg. "Alk and Ret Inhibitors Promote Hla Class I Antigen Presentation and Unmask New Antigens within the Tumor Immunopeptidome." *Cancer Immunology Research* 7, no. 12 (2019): 1984-97.
9. Pyo, Kyoung-Ho, Sun Min Lim, Chae-Won Park, Ha-Ni Jo, Jae Hwan Kim, Mi-Ran Yun, Dohee Kim, Chun-Feng Xin, Wongeun Lee, Bianca Gheorghiu, Min Hee Hong, Hye Ryun Kim, Hyo Sup Shim, Mi Jang, Sung Sook Lee, and Byoung Chul Cho. "Comprehensive Analyses of Immunodynamics and Immunoreactivity in Response to Treatment in Alk-Positive Non-Small-Cell Lung Cancer." *Journal for ImmunoTherapy of Cancer* 8, no. 2 (2020): e000970.
10. Vladimer, Gregory I., Berend Snijder, Nikolaus Krall, Johannes W. Bigenzahn, Kilian V. M. Huber, Charles-Hugues Lardeau, Kumar Sanjiv, Anna Ringler, Ulrika Warpman Berglund, Monika Sabler, Oscar Lopez de la Fuente, Paul Knöbl, Stefan Kubicek, Thomas Helleday, Ulrich Jäger, and Giulio Superti-Furga. "Global Survey of the Immunomodulatory Potential of Common Drugs." *Nat Chem Biol* 13, no. 6 (2017): 681-90.

11. Liu, P., L. Zhao, J. Pol, S. Levesque, A. Petrazzuolo, C. Pfirschke, C. Engblom, S. Rickelt, T. Yamazaki, K. Iribarren, L. Senovilla, L. Bezu, E. Vacchelli, V. Sica, A. Melis, T. Martin, L. Xia, H. Yang, Q. Li, J. Chen, S. Durand, F. Aprahamian, D. Lefevre, S. Broutin, A. Paci, A. Bongers, V. Minard-Colin, E. Tartour, L. Zitvogel, L. Apetoh, Y. Ma, M. J. Pittet, O. Kepp, and G. Kroemer. "Crizotinib-Induced Immunogenic Cell Death in Non-Small Cell Lung Cancer." *Nat Commun* 10, no. 1 (2019): 1486.
12. Liu, Peng, Liwei Zhao, Oliver Kepp, and Guido Kroemer. "Crizotinib - a Tyrosine Kinase Inhibitor That Stimulates Immunogenic Cell Death." *Oncoimmunology* 8, no. 7 (2019): 1596652-52.
13. Lin, J. J., E. Chin, B. Y. Yeap, L. A. Ferris, V. Kamesan, I. T. Lennes, L. V. Sequist, R. S. Heist, M. Mino-Kenudson, J. F. Gainor, and A. T. Shaw. "Increased Hepatotoxicity Associated with Sequential Immune Checkpoint Inhibitor and Crizotinib Therapy in Patients with Non-Small Cell Lung Cancer." *J Thorac Oncol* 14, no. 1 (2019): 135-40.
14. Dummer, Reinhard, Egle Ramelyte, Sabrina Schindler, Olaf Thürigen, Mitchell P. Levesque, and Peter Koelblinger. "Mek Inhibition and Immune Responses in Advanced Melanoma." *Oncoimmunology* 6, no. 8 (2017): e1335843.
15. Yarchoan, Mark, Aditya A. Mohan, Lauren Dennison, Teena Vithayathil, Amanda Ruggieri, Gregory B. Lesinski, Todd D. Armstrong, Nilofer S. Azad, and Elizabeth M. Jaffee. "Mek Inhibition Suppresses B Regulatory Cells and Augments Anti-Tumor Immunity." *PloS one* 14, no. 10 (2019): e0224600-e00.
16. Ebert, P. J. R., J. Cheung, Y. Yang, E. McNamara, R. Hong, M. Moskalenko, S. E. Gould, H. Maecker, B. A. Irving, J. M. Kim, M. Belvin, and I. Mellman. "Map Kinase Inhibition Promotes T Cell and Anti-Tumor Activity in Combination with Pd-L1 Checkpoint Blockade." *Immunity* 44, no. 3 (2016): 609-21.
17. Bendell, J., F. Ciardiello, J. Tabernero, N. Tebbutt, C. Eng, M. Di Bartolomeo, A. Falcone, M. Fakih, M. Kozloff, N. Segal, A. Sobrero, Y. Shi, L. Roberts, Y. Yan, I. Chang, A. Uyei, and T. Kim. "Lba-004 - Efficacy and Safety Results from Imblaze370, a Randomised Phase Iii Study Comparing Atezolizumab+Cobimetinib and Atezolizumab Monotherapy Vs Regorafenib in Chemotherapy-Refractory Metastatic Colorectal Cancer." *Annals of Oncology* 29 (2018): v123.
18. Liu, Li, Patrick A. Mayes, Stephen Eastman, Hong Shi, Sapna Yadavilli, Tianqian Zhang, Jingsong Yang, Laura Seestaller-Wehr, Shu-Yun Zhang, Chris Hopson, Lyuben Tsvetkov, Junping Jing, Shu Zhang, James Smothers, and Axel Hoos. "The Braf and Mek Inhibitors Dabrafenib and Trametinib: Effects on Immune Function and in Combination with Immunomodulatory Antibodies Targeting Pd-1, Pd-L1, and Ctla-4." *Clinical Cancer Research* 21, no. 7 (2015): 1639-51.
19. Frederick, D. T., A. Piris, A. P. Cogdill, Z. A. Cooper, C. Lezcano, C. R. Ferrone, D. Mitra, A. Boni, L. P. Newton, C. Liu, W. Peng, R. J. Sullivan, D. P. Lawrence, F. S. Hodi, W. W. Overwijk, G. Lizée, G. F. Murphy, P. Hwu, K. T. Flaherty, D. E. Fisher, and J. A. Wargo. "Braf Inhibition Is Associated with Enhanced Melanoma Antigen Expression and a More Favorable Tumor Microenvironment in Patients with Metastatic Melanoma." *Clinical cancer research : an official journal of the American Association for Cancer Research* 19, no. 5 (2013): 1225-31.
20. Deken, Marcel A., Jules Gadiot, Ekaterina S. Jordanova, Ruben Lacroix, Melissa van Gool, Paula Kroon, Cristina Pineda, Marnix H. Geukes Foppen, Richard Scolyer, Ji-Ying Song, Inge Verbrugge, Christoph Hoeller, Reinhard Dummer, John B. A. G. Haanen, Georgina V. Long, and Christian U. Blank. "Targeting the Mapk and Pi3k Pathways in Combination with Pd1 Blockade in Melanoma." *Oncoimmunology* 5, no. 12 (2016): e1238557-e57.
21. Hu-Lieskovan, Siwen, Stephen Mok, Blanca Homet Moreno, Jennifer Tsoi, Lidia Robert, Lucas Goedert, Elaine M. Pinheiro, Richard C. Koya, Thomas G. Graeber, Begoña Comin-Anduix, and Antoni Ribas. "Improved Antitumor Activity of Immunotherapy with Braf and Mek Inhibitors in Braf(V600e) Melanoma." *Sci Transl Med* 7, no. 279 (2015): 279ra41-79ra41.
22. Proietti, Ilaria, Nevena Skroza, Simone Michelini, Alessandra Mambrin, Veronica Balduzzi, Nicoletta Bernardini, Anna Marchesiello, Ersilia Tolino, Salvatore Volpe, Patrizia Maddalena, Marco Di Fraia, Giorgio Mangino, Giovanna Romeo, and Concetta Potenza. "Braf Inhibitors: Molecular Targeting and Immunomodulatory Actions." *Cancers* 12, no. 7 (2020): 1823.
23. Vella, Laura J., Miles C. Andrews, Anupama Pasam, Katherine Woods, Andreas Behren, and Jonathan S. Cebon. "The Kinase Inhibitors Dabrafenib and Trametinib Affect Isolated Immune Cell Populations." *Oncoimmunology* 3, no. 7 (2014): e946367-e67.
24. Schilling, B., W. Sondermann, F. Zhao, K. G. Griewank, E. Livingstone, A. Sucker, H. Zelba, B. Weide, U. Trefzer, T. Wilhelm, C. Loquai, C. Berking, J. Hassel, K. C. Kähler, J. Utikal, P. Al Ghazal, R. Gutzmer, S. M. Goldinger, L. Zimmer, A. Paschen, U. Hillen, D. Schadendorf, and DeCog. "Differential Influence of Vemurafenib and Dabrafenib on Patients' Lymphocytes Despite Similar Clinical Efficacy in Melanoma."

Annals of oncology : official journal of the European Society for Medical Oncology 25, no. 3 (2014): 747-53.
25. Schilling, Bastian, and Annette Paschen. "Immunological Consequences of Selective Braf Inhibitors in Malignant Melanoma: Neutralization of Myeloid-Derived Suppressor Cells." *Oncoimmunology* 2, no. 8 (2013): e25218-e18.
26. Sapkota, Bishu, Charles E. Hill, and Brian P. Pollack. "Vemurafenib Enhances Mhc Induction in Braf(V600e) Homozygous Melanoma Cells." *Oncoimmunology* 2, no. 1 (2013): e22890-e90.
27. Jin Suh, Koung, Ji Hea Sung, Jin Won Kim, Song-Hee Han, Hye Seung Lee, Ahrum Min, Mi Hyun Kang, Ji Eun Kim, Ji-Won Kim, Se Hyun Kim, Jeong-Ok Lee, Yu Jung Kim, Keun-Wook Lee, Soo-Mee Bang, Seock-Ah Im, and Jong Seok Lee. "Egfr or Her2 Inhibition Modulates the Tumor Microenvironment by Suppression of Pd-L1 and Cytokines Release." *Oncotarget* 8, no. 38 (2017).
28. Venugopalan, Abhilash, Min-Jung Lee, Gang Niu, José Medina-Echeverz, Yusuke Tomita, Martin J. Lizak, Constance M. Cultraro, Robert Mark Simpson, Xiaoyuan Chen, Jane B. Trepel, and Udayan Guha. "Egfr-Targeted Therapy Results in Dramatic Early Lung Tumor Regression Accompanied by Imaging Response and Immune Infiltration in Egfr Mutant Transgenic Mouse Models." *Oncotarget* 7, no. 34 (2016).
29. Lizotte, P. H., R. L. Hong, T. A. Luster, M. E. Cavanaugh, L. J. Taus, S. Wang, A. Dhaneshwar, N. Mayman, A. Yang, M. Kulkarni, L. Badalucco, E. Fitzpatrick, H. F. Kao, M. Kuraguchi, M. Bittinger, P. T. Kirschmeier, N. S. Gray, D. A. Barbie, and P. A. Janne. "A High-Throughput Immune-Oncology Screen Identifies Egfr Inhibitors as Potent Enhancers of Antigen-Specific Cytotoxic T-Lymphocyte Tumor Cell Killing." *Cancer Immunol Res* 6, no. 12 (2018): 1511-23.
30. Jia, Yijun, Xuefei Li, Tao Jiang, Sha Zhao, Chao Zhao, Limin Zhang, Xiaozhen Liu, Jinpeng Shi, Meng Qiao, Jiawei Luo, Sangtian Liu, Ruoshuang Han, Chunxia Su, Shengxiang Ren, and Caicun Zhou. "Egfr-Targeted Therapy Alters the Tumor Microenvironment in Egfr-Driven Lung Tumors: Implications for Combination Therapies." *International Journal of Cancer* 145, no. 5 (2019): 1432-44.
31. Hannesdottir, L., P. Tymoszuk, N. Parajuli, M. H. Wasmer, S. Philipp, N. Daschil, S. Datta, J. B. Koller, C. H. Tripp, P. Stoitzner, E. Muller-Holzner, G. J. Wiegers, V. Sexl, A. Villunger, and W. Doppler. "Lapatinib and Doxorubicin Enhance the Stat1-Dependent Antitumor Immune Response." *European journal of immunology* (2013).
32. Booth, L., J. L. Roberts, A. Poklepovic, F. Avogadri-Connors, R. E. Cutler, A. S. Lalani, and P. Dent. "Hdac Inhibitors Enhance Neratinib Activity and When Combined Enhance the Actions of an Anti-Pd-1 Immunomodulatory Antibody in Vivo." *Oncotarget* 8, no. 52 (2017): 90262-77.
33. HOLUBEC, LUBOS, JIRI POLIVKA, MARTIN SAFANDA, MICHAL KARAS, and VACLAV LISKA. "The Role of Cetuximab in the Induction of Anticancer Immune Response in Colorectal Cancer Treatment." *Anticancer Research* 36, no. 9 (2016): 4421-26.
34. Botta, C., E. Bestoso, S. Apollinari, M. G. Cusi, P. Pastina, A. Abbruzzese, P. Sperlongano, G. Misso, M. Caraglia, P. Tassone, P. Tagliaferri, and P. Correale. "Immune-Modulating Effects of the Newest Cetuximab-Based Chemoimmunotherapy Regimen in Advanced Colorectal Cancer Patients." *J Immunother* 35, no. 5 (2012): 440-7.
35. Nordstrom, J. L., S. Gorlatov, W. Zhang, Y. Yang, L. Huang, S. Burke, H. Li, V. Ciccarone, T. Zhang, J. Stavenhagen, S. Koenig, S. J. Stewart, P. A. Moore, S. Johnson, and E. Bonvini. "Anti-Tumor Activity and Toxicokinetics Analysis of Mgah22, an Anti-Her2 Monoclonal Antibody with Enhanced Fcγ Receptor Binding Properties." *Breast Cancer Research* 13, no. 6 (2011).
36. Nordstrom, Jeffrey L., John Muth, Courtney L. Erskine, Catherine Sanders, Erik C. Yusko, Ryan O Emerson, Min-Jung Lee, Sunmin Lee, Jane B Trepel, Seock-Ah Im, Yung-Jue Bang, Giuseppe Giaccone, Todd Michael Bauer, Howard A. Burris, Jan E. Baughman, Edwin P. Rock, Paul A. Moore, Ezio Bonvini, and Keith L Knutson. "High Frequency of Her2-Specific Immunity Observed in Patients (Pts) with Her2+ Cancers Treated with Margetuximab (M), an Fc-Enhanced Anti-Her2 Monoclonal Antibody (Mab)." *Journal of Clinical Oncology* 37, no. 15_suppl (2019): 1030-30.
37. Genova, Carlo, and Fred R. Hirsch. "Clinical Potential of Necitumumab in Non-Small Cell Lung Carcinoma." *OncoTargets and therapy* 9 (2016): 5427-37.
38. Trivedi, Sumita, Raghvendra M. Srivastava, Fernando Concha-Benavente, Soldano Ferrone, Tatiana M. Garcia-Bates, Jing Li, and Robert L. Ferris. "Anti-Egfr Targeted Monoclonal Antibody Isotype Influences Antitumor Cellular Immunity in Head and Neck Cancer Patients." *Clinical cancer research : an official journal of the American Association for Cancer Research* 22, no. 21 (2016): 5229-37.

39. Di Modica, Martina, Elda Tagliabue, and Tiziana Triulzi. "Predicting the Efficacy of Her2-Targeted Therapies: A Look at the Host." *Disease Markers* 2017 (2017): 7849108.
40. Pascual, Julio, Stefan P. Berger, Oliver Witzke, Helio Tedesco, Shamkant Mulgaonkar, Yasir Qazi, Steven Chadban, Federico Oppenheimer, Claudia Sommerer, Rainer Oberbauer, Yoshihiko Watarai, Christophe Legendre, Franco Citterio, Mitchell Henry, Titte R. Srinivas, Wen-Lin Luo, AnaMaria Marti, Peter Bernhardt, and Flavio Vincenti. "Everolimus with Reduced Calcineurin Inhibitor Exposure in Renal Transplantation." *Journal of the American Society of Nephrology* 29, no. 7 (2018): 1979-91.
41. Liu, Jinyu, Dong Liu, Juan Li, Lan Zhu, Chengliang Zhang, Kai Lei, Qiling Xu, and Ruxu You. "Efficacy and Safety of Everolimus for Maintenance Immunosuppression of Kidney Transplantation: A Meta-Analysis of Randomized Controlled Trials." *PloS one* 12, no. 1 (2017): e0170246.
42. Templeton, A. J., V. Dutoit, R. Cathomas, C. Rothermundt, D. Bärtschi, C. Dröge, O. Gautschi, M. Borner, E. Fechter, F. Stenner, R. Winterhalder, B. Müller, R. Schiess, P. J. Wild, J. H. Rüschoff, G. Thalmann, P. Y. Dietrich, R. Aebersold, D. Klingbiel, and S. Gillessen. "Phase 2 Trial of Single-Agent Everolimus in Chemotherapy-Naive Patients with Castration-Resistant Prostate Cancer (Sakk 08/08)." *Eur Urol* 64, no. 1 (2013): 150-8.
43. Pradier, Amandine, Maria Papaserafeim, Ning Li, Anke Rietveld, Charlotte Kaestel, Lyssia Gruaz, Cédric Vonarburg, Rolf Spirig, Gisella L. Puga Yung, and Jörg D. Seebach. "Small-Molecule Immunosuppressive Drugs and Therapeutic Immunoglobulins Differentially Inhibit Nk Cell Effector Functions in Vitro." *Frontiers in immunology* 10 (2019): 556-56.
44. Huijts, C. M., S. M. Lougheed, Z. Bodalal, C. M. van Herpen, P. Hamberg, M. Tascilar, J. B. Haanen, H. M. Verheul, T. D. de Gruijl, and H. J. van der Vliet. "The Effect of Everolimus and Low-Dose Cyclophosphamide on Immune Cell Subsets in Patients with Metastatic Renal Cell Carcinoma: Results from a Phase I Clinical Trial." *Cancer immunology, immunotherapy : CII* 68, no. 3 (2019): 503-15.
45. Wang, Y., X. Y. Wang, J. R. Subjeck, P. A. Shrikant, and H. L. Kim. "Temsirolimus, an Mtor Inhibitor, Enhances Anti-Tumour Effects of Heat Shock Protein Cancer Vaccines." *Br J Cancer* 104, no. 4 (2011): 643-52.
46. Stehle, Franziska, Kristin Schulz, Corinna Fahldieck, Jana Kalich, Rudolf Lichtenfels, Dagmar Riemann, and Barbara Seliger. "Reduced Immunosuppressive Properties of Axitinib in Comparison with Other Tyrosine Kinase Inhibitors." *The Journal of biological chemistry* 288, no. 23 (2013): 16334-47.
47. Kwilas, Anna R., Andressa Ardiani, Renee N. Donahue, Dana T. Aftab, and James W. Hodge. "Dual Effects of a Targeted Small-Molecule Inhibitor (Cabozantinib) on Immune-Mediated Killing of Tumor Cells and Immune Tumor Microenvironment Permissiveness When Combined with a Cancer Vaccine." *Journal of Translational Medicine* 12 (2014): 294-94.
48. Kwilas, Anna R., Renee N. Donahue, Kwong Y. Tsang, and James W. Hodge. "Immune Consequences of Tyrosine Kinase Inhibitors That Synergize with Cancer Immunotherapy." *Cancer cell & microenvironment* 2, no. 1 (2015): e677.
49. Li, Hui, Chia-Wei Li, Xiaoqiang Li, Qingqing Ding, Lei Guo, Shuang Liu, Chunxiao Liu, Chien-Chen Lai, Jung-Mao Hsu, Qiongzhu Dong, Weiya Xia, Jennifer L. Hsu, Hirohito Yamaguchi, Yi Du, Yun-Ju Lai, Xian Sun, Paul B. Koller, Qinghai Ye, and Mien-Chie Hung. "Met Inhibitors Promote Liver Tumor Evasion of the Immune Response by Stabilizing Pdl1." *Gastroenterology* 156, no. 6 (2019): 1849-61.e13.
50. Palakurthi, Sangeetha, Mari Kuraguchi, Sima J. Zacharek, Enrique Zudaire, Wei Huang, Dennis M. Bonal, Jeffrey Liu, Abha Dhaneshwar, Kristin DePeaux, Martha R. Gowaski, Dyane Bailey, Samuel N. Regan, Elena Ivanova, Catherine Ferrante, Jessie M. English, Aditya Khosla, Andrew H. Beck, Julie A. Rytlewski, Catherine Sanders, Sylvie Laquerre, Mark A. Bittinger, Paul T. Kirschmeier, Kathryn Packman, Pasi A. Janne, Christopher Moy, Kwok-Kin Wong, Raluca I. Verona, and Matthew V. Lorenzi. "The Combined Effect of Fgfr Inhibition and Pd-1 Blockade Promotes Tumor-Intrinsic Induction of Antitumor Immunity." *Cancer Immunology Research* 7, no. 9 (2019): 1457-71.
51. Balachandran, V. P., M. J. Cavnar, S. Zeng, Z. M. Bamboat, L. M. Ocuin, H. Obaid, E. C. Sorenson, R. Popow, C. Ariyan, F. Rossi, P. Besmer, T. Guo, C. R. Antonescu, T. Taguchi, J. Yuan, J. D. Wolchok, J. P. Allison, and R. P. DeMatteo. "Imatinib Potentiates Antitumor T Cell Responses in Gastrointestinal Stromal Tumor through the Inhibition of Ido." *Nat Med* 17, no. 9 (2011): 1094-100.
52. Tan, Y., M. T. Garcia-Buitrago, J. C. Trent, and A. E. Rosenberg. "The Immune System and Gastrointestinal Stromal Tumor: A Wealth of Opportunities." *Curr Opin Oncol* 27, no. 4 (2015): 338-42.
53. Wolf, D., H. Tilg, H. Rumpold, G. Gastl, and A. M. Wolf. "The Kinase Inhibitor Imatinib--an Immunosuppressive Drug?" *Curr Cancer Drug Targets* 7, no. 3 (2007): 251-8.

54. van Dongen, M., N. D. Savage, E. S. Jordanova, I. H. Briaire-de Bruijn, K. V. Walburg, T. H. Ottenhoff, P. C. Hogendoorn, S. H. van der Burg, H. Gelderblom, and T. van Hall. "Anti-Inflammatory M2 Type Macrophages Characterize Metastasized and Tyrosine Kinase Inhibitor-Treated Gastrointestinal Stromal Tumors." *International journal of cancer. Journal international du cancer* 127, no. 4 (2010): 899-909.
55. Taylor, J. R., N. Brownlow, J. Domin, and N. J. Dibb. "Fms Receptor for M-Csf (Csf-1) Is Sensitive to the Kinase Inhibitor Imatinib and Mutation of Asp-802 to Val Confers Resistance." *Oncogene* 25, no. 1 (2006): 147-51.
56. Lin, Y. Y., C. T. Tan, C. W. Chen, D. L. Ou, A. L. Cheng, and C. Hsu. "Immunomodulatory Effects of Current Targeted Therapies on Hepatocellular Carcinoma: Implication for the Future of Immunotherapy." *Semin Liver Dis* 38, no. 4 (2018): 379-88.
57. Kato, Yu, Kimiyo Tabata, Takayuki Kimura, Ayako Yachie-Kinoshita, Yoichi Ozawa, Kazuhiko Yamada, Junichi Ito, Sho Tachino, Yusaku Hori, Masahiro Matsuki, Yukiko Matsuoka, Samik Ghosh, Hiroaki Kitano, Kenichi Nomoto, Junji Matsui, and Yasuhiro Funahashi. "Lenvatinib Plus Anti-Pd-1 Antibody Combination Treatment Activates Cd8+ T Cells through Reduction of Tumor-Associated Macrophage and Activation of the Interferon Pathway." *PloS one* 14, no. 2 (2019): e0212513-e13.
58. Kato, Y., X. Bao, S. Macgrath, K. Tabata, Y. Hori, S. Tachino, M. Matijevici, Y. Funahashi, and J. Matsui. "Lenvatinib Mesilate (Len) Enhanced Antitumor Activity of a Pd-1 Blockade Agent by Potentiating Th1 Immune Response." *Annals of Oncology* 27 (2016): vi1.
59. Keohane, Clodagh, Shahram Kordasti, Thomas Seidl, Pilar Perez Abellan, Nicholas S. B. Thomas, Claire N. Harrison, Donal P. McLornan, and Ghulam J. Mufti. "Jak Inhibition Induces Silencing of T Helper Cytokine Secretion and a Profound Reduction in T Regulatory Cells." *Br J Haematol* 171, no. 1 (2015): 60-73.
60. Pitroda, Sean P., Melinda E. Stack, Gene-Fu Liu, Sui-Sui Song, Lucy Chen, Hua Liang, Akash D. Parekh, Xiaona Huang, Paul Roach, Mitchell C. Posner, Ralph R. Weichselbaum, and Nikolai N. Khodarev. "Jak2 Inhibitor Sar302503 Abrogates Pd-L1 Expression and Targets Therapy-Resistant Non–Small Cell Lung Cancers." *Molecular Cancer Therapeutics* 17, no. 4 (2018): 732-39.
61. Hao, Yansheng, Bjoern Chapuy, Stefano Monti, Heather H. Sun, Scott J. Rodig, and Margaret A. Shipp. "Selective Jak2 Inhibition Specifically Decreases Hodgkin Lymphoma and Mediastinal Large B-Cell Lymphoma Growth in Vitro and in Vivo." *Clinical cancer research : an official journal of the American Association for Cancer Research* 20, no. 10 (2014): 2674-83.
62. Zizzari, I. G., C. Napoletano, A. Botticelli, S. Caponnetto, F. Calabrò, A. Gelibter, A. Rughetti, I. Ruscito, H. Rahimi, E. Rossi, G. Schinzari, P. Marchetti, and M. Nuti. "Tk Inhibitor Pazopanib Primes Dcs by Downregulation of the B-Catenin Pathway." *Cancer Immunol Res* 6, no. 6 (2018): 711-22.
63. Dammeijer, Floris, Lysanne A. Lievense, Margaretha E. Kaijen-Lambers, Menno van Nimwegen, Koen Bezemer, Joost P. Hegmans, Thorbald van Hall, Rudi W. Hendriks, and Joachim G. Aerts. "Depletion of Tumor-Associated Macrophages with a Csf-1r Kinase Inhibitor Enhances Antitumor Immunity and Survival Induced by Dc Immunotherapy." *Cancer Immunology Research* 5, no. 7 (2017): 535-46.
64. Mok, S., R. C. Koya, C. Tsui, J. Xu, L. Robert, L. Wu, T. Graeber, B. L. West, G. Bollag, and A. Ribas. "Inhibition of Csf-1 Receptor Improves the Antitumor Efficacy of Adoptive Cell Transfer Immunotherapy." *Cancer Research* 74, no. 1 (2014): 153-61.
65. Shigeta, Kohei, Aya Matsui, Hiroto Kikuchi, Sebastian Klein, Emilie Mamessier, Ivy X. Chen, Shuichi Aoki, Shuji Kitahara, Koetsu Inoue, Ayako Shigeta, Tai Hato, Rakesh R. Ramjiawan, Daniel Staiculescu, Dieter Zopf, Lukas Fiebig, Gabriela S. Hobbs, Alexander Quaas, Simona Dima, Irinel Popescu, Peigen Huang, Lance L. Munn, Mark Cobbold, Lipika Goyal, Andrew X. Zhu, Rakesh K. Jain, and Dan G. Duda. "Regorafenib Combined with Pd1 Blockade Increases Cd8 T-Cell Infiltration by Inducing Cxcl10 Expression in Hepatocellular Carcinoma." *Journal for ImmunoTherapy of Cancer* 8, no. 2 (2020): e001435.
66. Arai, Hiroyuki, Francesca Battaglin, Jingyuan Wang, Jae Ho Lo, Shivani Soni, Wu Zhang, and Heinz-Josef Lenz. "Molecular Insight of Regorafenib Treatment for Colorectal Cancer." *Cancer treatment reviews* 81 (2019): 101912-12.
67. Elli, E. M., C. Baratè, F. Mendicino, F. Palandri, and G. A. Palumbo. "Mechanisms Underlying the Anti-Inflammatory and Immunosuppressive Activity of Ruxolitinib." *Front Oncol* 9 (2019): 1186.
68. Kenderian, Saad S, Marco Ruella, Olga Shestova, Miriam Y Kim, Michael Klichinsky, Fang Chen, Natalka Kengle, Simon Francis Lacey, J. Joseph Melenhorst, Carl H June, and Saar I. Gill. "Ruxolitinib Prevents Cytokine Release Syndrome after Cart Cell Therapy without Impairing the Anti-Tumor Effect in a Xenograft Model." *Blood* 128, no. 22 (2016): 652-52.

69. Lu, Chunwan, Asif Talukder, Natasha M. Savage, Nagendra Singh, and Kebin Liu. "Jak-Stat-Mediated Chronic Inflammation Impairs Cytotoxic T Lymphocyte Activation to Decrease Anti-Pd-1 Immunotherapy Efficacy in Pancreatic Cancer." *Oncoimmunology* 6, no. 3 (2017): e1291106-e06.
70. Farsaci, Benedetto, Jack P. Higgins, and James W. Hodge. "Consequence of Dose Scheduling of Sunitinib on Host Immune Response Elements and Vaccine Combination Therapy." *International Journal of Cancer* 130, no. 8 (2012): 1948-59.
71. Desar, I. M., J. H. Jacobs, C. A. Hulsbergen-vandeKaa, W. J. Oyen, P. F. Mulders, W. T. van der Graaf, G. J. Adema, C. M. van Herpen, and I. J. de Vries. "Sorafenib Reduces the Percentage of Tumour Infiltrating Regulatory T Cells in Renal Cell Carcinoma Patients." *International journal of cancer. Journal international du cancer* 129, no. 2 (2011): 507-12.
72. Xin, H., C. Zhang, A. Herrmann, Y. Du, R. Figlin, and H. Yu. "Sunitinib Inhibition of Stat3 Induces Renal Cell Carcinoma Tumor Cell Apoptosis and Reduces Immunosuppressive Cells." *Cancer Research* 69, no. 6 (2009): 2506-13.
73. Ozao-Choy, Junko, Ge Ma, Johnny Kao, George X. Wang, Marcia Meseck, Max Sung, Myron Schwartz, Celia M. Divino, Ping-Ying Pan, and Shu-Hsia Chen. "The Novel Role of Tyrosine Kinase Inhibitor in the Reversal of Immune Suppression and Modulation of Tumor Microenvironment for Immune-Based Cancer Therapies." *Cancer Research* 69, no. 6 (2009): 2514-22.
74. Adotevi, O., H. Pere, P. Ravel, N. Haicheur, C. Badoual, N. Merillon, J. Medioni, S. Peyrard, S. Roncelin, V. Verkarre, A. Mejean, W. H. Fridman, S. Oudard, and E. Tartour. "A Decrease of Regulatory T Cells Correlates with Overall Survival after Sunitinib-Based Antiangiogenic Therapy in Metastatic Renal Cancer Patients." *J Immunother* 33, no. 9 (2010): 991-8.
75. Finke, James H., Brian Rini, Joanna Ireland, Patricia Rayman, Amy Richmond, Ali Golshayan, Laura Wood, Paul Elson, Jorge Garcia, Robert Dreicer, and Ronald Bukowski. "Sunitinib Reverses Type-1 Immune Suppression and Decreases T-Regulatory Cells in Renal Cell Carcinoma Patients." *Clinical Cancer Research* 14, no. 20 (2008): 6674-82.
76. Tada, Y., Y. Togashi, D. Kotani, T. Kuwata, E. Sato, A. Kawazoe, T. Doi, H. Wada, H. Nishikawa, and K. Shitara. "Targeting Vegfr2 with Ramucirumab Strongly Impacts Effector/ Activated Regulatory T Cells and Cd8(+) T Cells in the Tumor Microenvironment." *J Immunother Cancer* 6, no. 1 (2018): 106.
77. Herbst, Roy S., Hendrik-Tobias Arkenau, Rafael Santana-Davila, Emiliano Calvo, Luis Paz-Ares, Philippe A. Cassier, Johanna Bendell, Nicolas Penel, Matthew G. Krebs, Juan Martin-Liberal, Nicolas Isambert, Andres Soriano, Martin Wermke, Jennifer Cultrera, Ling Gao, Ryan C. Widau, Gu Mi, Jin Jin, David Ferry, Charles S. Fuchs, Daniel P. Petrylak, and Ian Chau. "Ramucirumab Plus Pembrolizumab in Patients with Previously Treated Advanced Non-Small-Cell Lung Cancer, Gastro-Oesophageal Cancer, or Urothelial Carcinomas (Jvdf): A Multicohort, Non-Randomised, Open-Label, Phase 1a/B Trial." *The Lancet Oncology* 20, no. 8 (2019): 1109-23.

Table 2. Ongoing clinical trials of kinase inhibitors approved for breast cancer treatment in combination with immunotherapy. Only the kinase inhibitors and the immunotherapeutic agents that are combined are listed.

Kinase inhibitor	Target	Immunotherapy	Phase	Subtype	Identifier
**CDK4/6 inhibitor**					
Abemaciclib	CDK4/6	Pembrolizumab	Ib	HR+(A/M)	NCT02779751
Abemaciclib	CDK4/6	LY3300054	Ia/b	HR+HER2-	NCT02791334
Abemaciclib	CDK4/6	Atezolizumab	Ib/II	HR+HER2-(A/M)	NCT03280563
Palbociclib	CDK4/6	Avelumab	Ib	AR+ER-HER-	NCT04360941
Palbociclib	CDK4/6	Avelumab	II	ER+	NCT03573648
Palbociclib	CDK4/6	Pembrolizumab	II	ER+(M) HER2-	NCT02778685
Palbociclib	CDK4/6	Avelumab	II	ER+(M) HER2-	NCT03147287
Palbociclib	CDK4/6	Nivolumab	II	ER+/HER2-	NCT04075604
Ribociclib	CDK4/6	Spartalizumab	I	ER+(M) HER2-	NCT03294694
**EGFR family inhibitors**					
Trastuzumab	HER2	TPIV100 vaccine	II	HER2+,stage II-III	NCT04197687
Trastuzumab	HER2	Durvalumab	Ib	HER2+(M)	NCT02649686*
Trastuzumab	HER2	Pembrolizumab	Ib/II	HER2+(M)	NCT04512261
Trastuzumab	HER2	Avelumab	II	HER2+(M)	NCT03414658
Trastuzumab	HER2	Envafolimab	II	HER2+(M)	NCT04034823
Trastuzumab, Tucatinib	HER2	Pembrolizumab	Ib/II	HER2+(M)	NCT04512261
Trastuzumab emtansine	HER2	Pembrolizumab	I	HER2+(M)	NCT03032107
Trastuzumab emtansine	HER2	Atezolizumab	II	HER2+(LA/M)	NCT02924883
Trastuzumab emtansine	HER2	Atezolizumab	III	HER2+,PDL1+ (LA/M)	NCT04740918
Trastuzumab deruxtecan	HER2	Durvalumab	Ib	HER2-low (A/M)	NCT04556773
Trastuzumab deruxtecan	HER2	Durvalumab	Ib/II	HER2+ (M)	NCT04538742
Trastuzumab deruxtecan	HER2	Nivolumab	Ib	HER2+(A/M)	NCT03523572
Trastuzumab, pertuzumab	HER2	Pembrolizumab	II	HER2+, LN positive	NCT03747120
Trastuzumab, pertuzumab	HER2	Atezolizumab	II	HER2 amplified	NCT03894007
Trastuzumab, pertuzumab	HER2	Atezolizumab	Ib	HER2+/-	NCT02605915
Trastuzumab, pertuzumab	HER2	Atezolizumab	III	HER2+ early	NCT03595592
Trastuzumab, pertuzumab	HER2	Atezolizumab	II	HER2+	NCT03125928
Trastuzumab, pertuzumab	HER2	Atezolizumab	III	HER2+ early	NCT03726879
Trastuzumab, pertuzumab	HER2	TAA vaccine	II	HER2+	NCT04329065
Trastuzumab, pertuzumab	HER2	Atezolizumab	III	HER2+(M)	NCT03199885
Trastuzumab, pertuzumab	HER2	Durvalumab	II	HER2+	NCT03820141
Trastuzumab, pertuzumab	HER2	IFNγ	I/II	HER2+	NCT03112590
Trastuzumab, pertuzumab	HER2	DC vaccine	I	HER2+	NCT03387553
Pertuzumab	HER2	Atezolizumab	II	HER2+	NCT03894007
Pertuzumab, ABP 980	HER2	Pembrolizumab	II	HER2+	NCT03988036
MEK inhibitors					
Binimetinib	MEK	Avelumab	II	TNBC	NCT03971409
Binimetinib	MEK	Pembrolizumab	Ib	all	NCT02779751
Binimetinib	MEK	Pembrolizumab	I/II	TNBC(LA/M)	NCT03106415
Cobimetinib	MEK	Atezolizumab	II	TNBC(M)	NCT02322814
Cobimetinib	MEK	Atezolizumab	I/II	ER+ Stage IV	NCT03566485
Cobimetinib	MEK	Atezolizumab	II	Inflammatory BC	NCT03202316
Cobimetinib	MEK	Atezolizumab	II	ER+HER2-	NCT03395899
Trametinib	MEK	PDR001 (anti-PD-1)	Ib	TNBC	NCT02900664
**Others**					
BGB324	Axl	Pembrolizumab	II	TNBC(M)	NCT03184558
Capmatinib	MET	spartalizumab +LAG525	I	TNBC	NCT03742349
Cabozantinib	c-Met, VEGFR1/2/3, FLT3, TIE2, TRKB, Axl, RET	Atezolizumab	Ib	LABC, TNBC	NCT03170960
Cabozantinib	c-Met, VEGFR1/2/3,	Nivolumab	I	TNBC	NCT04514484

	FLT3, TIE2, TRKB, Axl, RET				
Cabozantinib	c-Met, VEGFR1/2/3, FLT3, TIE2, TRKB, Axl, RET	Nivolumab	II	TNBC(M)	NCT03316586
Lenvatinib	VEGFR2	Pembrolizumab	II	TNBC	NCT03797326
Lenvatinib	VEGFR2	Pembrolizumab	I	TNBC	NCT04427293
Ruxolitinib	JAK2	Pembrolizumab	I	HR-, TNBC(M)	NCT03012230
Everolimus	mTOR	Spartalizumab	Ib	TNBC	NCT02890069

Source: Clinicaltrials.gov. **P**, early phase 1/pilot. **(L)A/M**, (locally) advanced and/or metastatic. **LABC**, locally advanced. Breast cancer. **ABP 980**: trastuzumab biosimilar ABP980. * = completed. **LN**, lymph node. **HR**, hormone receptor
